# Respiratory muscle activity after spontaneous, neostigmine- or sugammadex-enhanced recovery of neuromuscular blockade: a double blind prospective randomized controlled trial

**DOI:** 10.1186/s12871-019-0863-y

**Published:** 2019-10-19

**Authors:** Tom Schepens, Koen Janssens, Sabine Maes, Davina Wildemeersch, Jurryt Vellinga, Philippe G. Jorens, Vera Saldien

**Affiliations:** 1 Department of Critical Care Medicine, Antwerp University Hospital, Edegem, Belgium; 2 Department of Neurosurgery, Antwerp University Hospital, Edegem, Belgium; 3 Department of Anesthesia, Antwerp University Hospital, Edegem, Belgium; 4 MedMen, Groningen, The Netherlands

**Keywords:** Neuromuscular blockade, Neuromuscular blocking agents, Sugammadex, Neostigmine, Respiratory outcome

## Abstract

**Background:**

The use of neostigmine after neuromuscular blockade (NMB) has been associated with postoperative respiratory complications. In previous studies, we found lower diaphragmatic activity after neostigmine reversal of NMB, compared to sugammadex. It is still unclear whether the adequate use of neostigmine guarantees normal respiratory muscle function after NMB. In this study, we wanted to assess the effect of commonly used degrees of NMB and their possible reversal strategies on respiratory muscle activity after the return of normal neuromuscular transmission.

**Methods:**

This is a randomized, controlled, parallel-group, single-centre, double-blind study in patients scheduled for intracranial surgery at a tertiary academic hospital in Belgium. All participants received target controlled propofol/remifentanil anesthesia and were randomized into one of five groups, receiving either a shallow NMB with no reversal (shallow/saline), a shallow NMB with sugammadex reversal (shallow/sugammadex), a moderate NMB with neostigmine reversal (moderate/neostigmine), a moderate NMB with sugammadex reversal (moderate/sugammadex), or a deep NMB with sugammadex reversal (deep/sugammadex). Primary and secondary outcome parameters were diaphragm and intercostal electromyographic (EMG) activity at the moment of resumed spontaneous breathing activity, defined as a maximal interval of 10 min after the first spontaneous breath.

**Results:**

For the five groups, a total of 55 patients could be included in the final analysis. Median time of spontaneous breathing analyzed was 5 min (IQR 3–9.5 min). Both the moderate/sugammadex and the moderate/neostigmine groups had lower levels of diaphragm EMG compared to the shallow/sugammadex group. The moderate/neostigmine group had lower levels of intercostal EMG activity compared to the shallow/saline group.

**Conclusions:**

In this study, the depth of neuromuscular blockade and type of reversal strategy impacts respiratory muscle activity at the moment of resumed spontaneous breathing and recovery of neuromuscular blockade. Both groups that received moderate NMB had lower levels of diaphragm EMG, compared to the shallow NMB group with sugammadex reversal. Compared to the shallow NMB group with no reversal, the moderate NMB with neostigmine reversal group had lower intercostal EMG activity.

**Trial registration:**

Clinicaltrials.gov NCT01962298 on October 9, 2013 and EudraCT 2013–001926-25 on October 10, 2013.

## Background

The use of neuromuscular blocking agents (NMBAs), their reversal agents and respiratory outcome after surgery is still the subject of research and debate [1, 2]. The use of neostigmine has been associated with postoperative respiratory complications in some studies [3–5], possibly due to paradoxical neostigmine-induced muscle weakness. Other studies could not confirm a clinical presence of acetylcholinesterase-induced muscle weakness [6]. Higher doses of neostigmine seem to worsen respiratory outcome than lower doses [7]. This seemingly paradoxical relation has different possible explanations. Inadequate, i.e. either too early or inappropriately dosed neostigmine administration is one of them. Secondly, neostigmine has a central depressant effect on nerve function, with possible respiratory muscle weakness as a result [8]. In previous studies, we found lower diaphragmatic activity after neostigmine reversal of a NMB, compared to sugammadex [9, 10]. In both studies, diaphragm electromyographic (EMG) activity was recorded in healthy volunteers during spontaneous breathing after NMB reversal. Lower diaphragm activity was associated with smaller tidal volumes and lower arterial oxygen levels. The alternative NMB reversal agent sugammadex, a selective relaxant binding agent, does not directly interact with the cholinergic system [11]. The use of sugammadex was associated with improved respiratory outcome in a cohort of elderly patients relative to neostigmine-reversed or non-reversed patients [12]. The recently published POPULAR study showed an increase in postoperative pulmonary complications after the use of NMBAs. Neither the use of reversal agents, nor the choice of neostigmine or sugammadex decreased the risk for these complications [13].

In this study, we wanted to assess the effect of commonly used degrees of NMB and their possible reversal strategies on respiratory muscle activity after the return of normal neuromuscular transmission. We used a transcutaneous electromyography (EMG) method to evaluate both diaphragm and intercostal muscle activity in a prospective, double-blind randomized controlled trial. We hypothesized that the use of neostigmine would lead to worse respiratory muscle activity.

## Methods

### Trial design, participants and randomization

This randomized, controlled, parallel-group, single-centre, double-blind study in patients scheduled for brain surgery was approved by the Ethics Committee of the Antwerp University Hospital (Reference: EC13/5/60) The data were collected at the Antwerp University Hospital, a tertiary academic hospital in Belgium. The trial was registered on clinicaltrials.gov with number NCT01962298 on October 9, 2013 and on EudraCT with reference 2013–001926-25 on October 10, 2013 before inclusion of the first patient in the trial. This trial adhered to the applicable CONSORT guidelines.

In total, 75 patients were enrolled after providing written informed consent. These patients were scheduled for elective neurosurgical procedures and screened for possible inclusion the day prior to their surgery. Individuals with BMI more than 30 kg/m^2^, history of smoking, medication known to interfere with neuromuscular blocking agents (e.g. corticosteroids, magnesium) [14], or current upper airway infection were excluded. The anesthetic protocol was kept very similar across all participants: we used target controlled propofol/remifentanil anesthesia with levels titrated by the attending anesthesiologist, blinded regarding group allocation. Unpremedicated participants were randomized into one of five groups on a 1–1–1-1-1 ratio by a study nurse who did not participate in any other part of the study: the first group received a single 0.6 mg/kg bolus of rocuronium at the beginning of anesthesia and no reversal (shallow/saline), the second group received a single 0.6 mg/kg bolus of rocuronium at the beginning of anesthesia and a 2 mg/kg sugammadex bolus at the end of the surgical procedure (shallow/sugammadex), even when TOF had already returned to ≥0.9. The third group received a 0.6 mg/kg bolus of rocuronium at the beginning of anesthesia and had their level of NMB titrated to a TOF of 1–2 with additional boluses of rocuronium during the surgery, and received a 50 μg/kg neostigmine with 10 μg/kg glycopyrrolate at the end of the surgery with a TOF of T2 (moderate/neostigmine). The fourth group had a similar moderate level of NMB as group 3, but received a 2 mg/kg dose of sugammadex at the end of the surgery, on TOF T2 (moderate/sugammadex). The fifth group received a deep NMB with a continuous rocuronium infusion titrated to PTC 1–2 and was reversed with sugammadex 4 mg/kg at the end of the procedure (deep/sugammadex). The male/female ratio was kept similar across all 5 groups. To maintain blinding, not the blinded attending anesthetist but a second anesthetist administered the muscle relaxants. We also ran a syringe with normal saline in groups without a deep NMB. All syringes were covered with tape to conceal the volume and color of the contents.

Noninvasive blood pressure was recorded every 5 min, and the heart rate, ECG, end-tidal carbon dioxide concentration and arterial oxygen saturation (SpO_2_) were continuously recorded. An intravenous cannula was inserted, and Plasmalyte® solution was infused at a rate of 80 ml/h. An arterial cannula was inserted into the radial artery after the induction of anesthesia. Body temperature was maintained by a forced warm air blanket. An auxiliary surface EMG (Dipha16, Inbiolab BV, DEMCON group, Groningen, The Netherlands) was recorded at the diaphragm, the abdomen and intercostal muscles. The electrodes for the EMG of the diaphragm (EMGdi) were placed at the posterior axillar line below the ribs and the midclavicular line below the ribs, and the intercostal muscles were recorded on the midclavicular line at the second intercostal space. The abdominal muscles were measured next to the midline at the umbilical level. These electrodes were used to measure in- and expiratory abdominal muscle activity and to separate diaphragm from abdominal muscle activity. The degree of neuromuscular blockade was continuously measured using accelerometry of the adductor pollicis muscle via ulnar nerve stimulation (TOF-watch SX; MIPM Mammendorfer Institut für Physik und Medizin GmbH, Munich, Germany). Anesthesia was induced via a propofol intravenous target-controlled infusion and the addition of remifentanil, the targets and corresponding depth on anesthesia was left to the discretion of the blinded anesthetist. The thumb was placed in a specially designed finger holder that allowed the thumb to move in a consistent direction. A surgical drape placed over the arm ensured that the blinded anesthetist could not see the thumb movements. Repetitive TOF stimulation was applied every 15 s to the ulnar nerve. TOF-Watch SX calibration was performed as previously described [8] and performed with the implemented algorithm in calibration mode two (Operating Manual TOF-Watch® SX), > 3 min after a 5-s 50-Hz tetanic stimulation, and was preceded by a 1-min repetitive TOF stimulation. After calibration, a 3- to 4-min repetitive TOF stimulation was performed before the administration of rocuronium. After baseline TOF measurements, rocuronium was injected. The TOF ratio was monitored, recorded and later analyzed using TOFMON software (Organon Laboratories Ltd., Dublin, Ireland). Normalized acceleromyographic TOF ratios were calculated by dividing the TOF ratio by the study participant’s baseline value.

Participants were ventilated with an inspired oxygen fraction of 0.4 to 0.5, with an end-tidal carbon dioxide target of 4.66 to 5.33 kPa and a positive end-expiratory pressure of 5 cmH_2_O. The patients were allowed to breathe spontaneously after their TOF had returned to 0.9, at which point the remifentanil and propofol infusions were discontinued. The participants’ tracheas were extubated as soon as they were awake and able to follow commands. The primary and secondary outcome parameters, a priori defined, were diaphragm and intercostal electromyographic activity respectively. These were recorded at the moment of resumed spontaneous breathing activity, defined as a maximal interval of 10 min after the first spontaneous breath.

### Measurements and data processing

**Fig. 1:**
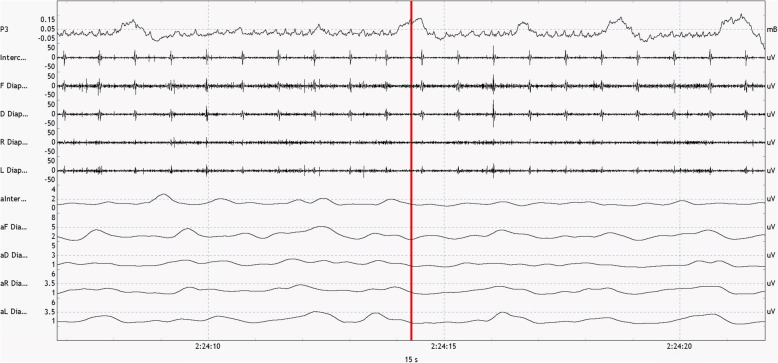
Sample of unfiltered EMG data. Top curve (“P3”) shows the pressure/flow signal, indicating inspiratory and expiratory flow. The following 5 curves show the raw EMG per set of leads for the diaphragm and the intercostal muscles. Bottom 5 show amplitudes of these raw signals. To obtain these data, electrodes are positioned bilaterally on the chest wall, on the mid-axillary and midclavicular lines just below the costal margins. The intercostal electrodes are positioned on the mid-clavicular line as well. Changes in electrical currents are measured in between a pair of electrodes, with a reference electrode positioned on the sternum. Currents flowing from the two right and left sided electrodes were labeled aR Dia and aL Dia, those in between the electrodes on the midclavicular line aF Dia, and those placed laterally aD Dia. The final data combined the signals from left and right

Data were sampled from the start of spontaneous breathing, defined as EMG activity or clinical chest wall or abdominal wall motion or flow detected on the anesthesia ventilator, until extubation. A sample of recorded EMG data is shown in Fig. 1. The last minute before extubation was later on not analyzed because of frequent coughing and the absence of quiet tidal breathing. We computed the median amplitude of EMG excursion from expiration to inspiration for the diaphragm and the intercostal muscles. After data collection, data were filtered (band-passed), processed and had a quality assessment. Amplitudes of EMG were calculated per patient starting from the baseline (tonic) level of diaphragm activity.

From these filtered data, we cut 30-s blocks for which the mean EMG amplitude per breath was extracted. Breath-by-breath analysis had the potential to generate skewed data, where patients breathing frequently would have a disproportionate amount of data points. We therefore cut the data into blocks to focus on the amplitude of breathing, regardless of the respiratory frequency. These blocks allowed us to have data that are both fit for analysis and adequately reflect inspiratory breathing effort: several breathing efforts are likely made in a 30 s interval, and longer intervals would mean fewer data points to use in the analysis. Furthermore, we a priori decided to use a maximal timeframe of 10 min per subject, as we otherwise would have risked that some patients who had a very long interval between start of spontaneous breathing and extubation had an excessive impact on the total amount of acquired data. These data, thus being a maximum of 20 values per patient, were used for the final analysis.

### Statistical analysis

Because this was the first study of this nature, we had no data available for sample size calculation. Instead, we chose to use at least the same number of volunteers as in a previous volunteer study that had proved satisfactory [9].

The data are expressed as median (IQR) unless otherwise stated. Comparisons were performed using one-way analysis of variance (nonparametric Kruskal – Wallis test), followed by a Mann-Whitney U test, when the *P* value was less than 0.05. We used the Bonferroni correction for multiplicity of testing each pairwise comparison. Categorical data are expressed as numbers (%). All statistical tests were two tailed, and a *P* value less than 0.05 was considered significant. Statistical analyses were performed using SPSS v24 for Mac.

## Results

**Fig. 2:**
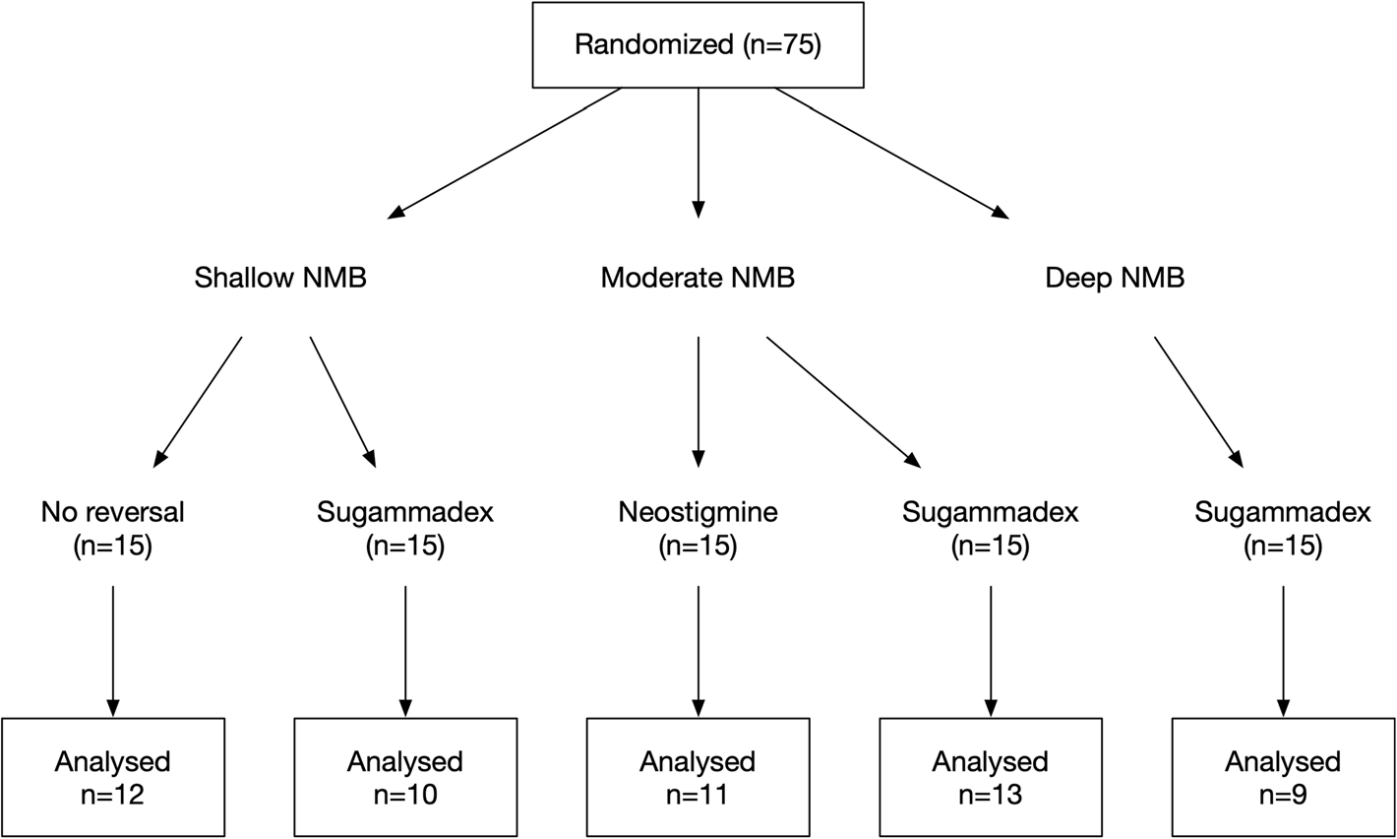
Simplified CONSORT flowchart. Legend: NMB: neuromuscular block

75 patients were successfully enrolled in this trial, and after final data processing 55 patients could be included in the final analysis. In total, 12 patients had a spontaneous recovery, 10 patients received neostigmine, and 33 received sugammadex. The CONSORT flowchart for the included patients and the reasons for non-analysis is shown in an additional file (Additional file 1), and Fig. 2 shows a simplified version. Table 1 displays the demographic characteristics of the included patients. There were no apparent clinically relevant differences in between the groups. Surgical times across the groups did not differ (*P* = 0.379). The patients with a moderate block and sugammadex reversal received a median of 40 mg of rocuronium after initial neuromuscular blockade, a similar amount compared to the moderate block and neostigmine reversal group, who had a median of 45 mg (*P* = 0.454).

**Table 1:** Demographics of included patients

Group	Patients analysed	Age (y)	BMI	Gender (M/F %)	ASA status (1/2)	Surgical time (m)	Reversal time (m)
Shallow/saline	12	54 (42–63)	26 (23–30)	50/50	4/8	162 (108–281)	
Shallow/sugammadex	10	51 (41–65)	27 (24–28)	27/73	3/6	168 (137–213)	
Moderate/neostigmine	11	56 (49–59)	25 (21–28)	40/60	2/9	178 (106–278)	17 (11–27)
Moderate/sugammadex	13	54 (30–68)	26 (24–28)	46/54	3/10	134 (114–198)	2 (2–2)
Deep/sugammadex	9	55 (41–63)	24 (21–27)	33/67	2/7	208 (177–234)	2 (2–3)

Age and BMI are displayed as median with interquartile range

Reversal time is the time from administration of neostigmine or sugammadex to TOF ≥ 90%

Surgical time is the from incision to wound closure

BMI: body mass index; ASA: American Society of Anesthesiologists

Median time of spontaneous breathing analysed was 5 min (IQR 3–9.5 min) and was similar among all groups (*P* = 0.14 independent samples median test). We were able to successfully reverse the neuromuscular block in all patients with the mentioned doses of the reversal agents. Every analysed patient started breathing after TOF had returned to ≥90%, and all patients were extubated ensuing a TOF value of ≥90%. One patient, who was excluded from analysis, started breathing before the TOF returned to 90%. Median diaphragm and intercostal EMG values are shown in Table 2. No patient needed re-intubation or non-invasive respiratory support after extubation, or desaturated < 90%.

**Table 2:** EMG values and time per group analyzed

Group	EMGdi (μV)	EMGintercostal (μV)	Time analysed (min)
Shallow/saline	3.7 (1.7–7.7)	2.4 (1.4–4.5)	3.0 (2.5–9.0)
Shallow/sugammadex	4.5 (2.1–8.0)	2.2 (1.2–4.7)	5.5 (3.5–7.0)
Moderate/neostigmine	3.2 (0.9–7.3)	1.5 (1.1–2.5)	4.0 (3.0–8.0)
Moderate/sugammadex	2.9 (1.3–5.3)	2.2 (1.3–4.5)	7.5 (5.0–10.0)
Deep/sugammadex	4.4 (1.9–7.5)	1.8 (1.1–3.7)	4.5 (3.0–10.0)

Data expressed as median with interquartile ranges

EMGdi = diaphragm electromyographic activity; EMGintercostal = intercostal electromyographic activity; μV = microvolt

Diaphragm EMG values were lower for the groups that received moderate levels of neuromuscular blockade, compared to the group with a shallow level of NMB and sugammadex administration. (*P* = 0.025 for moderate/neostigmine versus shallow/sugammadex and *P* = 0.009 for moderate/sugammadex versus shallow/sugammadex). In contrast, the intercostal EMG amplitude was lower for the moderate/neostigmine group, compared to the shallow/saline group (*P* = 0.019).

## Discussion

In this study, we present the diaphragm and intercostal EMG values as a parameter for inspiratory muscle activity after different types of neuromuscular blockade and reversal strategies. Interestingly, the patients that received a moderate level of neuromuscular blockade, reversed with neostigmine or sugammadex, had lower diaphragm EMG activity when breathing spontaneously at the end of the surgical procedure compared to the shallow/neostigmine group. The intercostal EMG amplitude was lower for the moderate/neostigmine group compared to the shallow/saline group (*P* = 0.019), only.

Interpretation of these results is not straightforward. Technical elements cannot be excluded to have had an effect on the outcome, reflected by the amount of drop-outs we experienced. However, as the patients were randomized these effects should have leveled out across all groups. Furthermore, meticulous attention to detail was present during data collection, including the correct placement of electrodes, the type of electrodes used, and quality monitoring during the recording. Quality of the data and band filtering for artifacts was performed before data analysis by Jurryt Vellinga and Leo A. Van Eykern. Different types of neuromuscular blockade and reversal strategies can both have an impact on respiratory activity, and the use of neuromuscular blockade by itself could impact respiratory outcome [13]. These results thus represent the effect of neuromuscular blockade as much as it reflects the reversal strategies, as the NMBA dose is an independent risk factor for adverse outcomes [15].

The effects of residual neuromuscular blockade are substantial and extensively discussed [16–18]. For this reason, in our study, all data was collected after the return of the TOFR to at least 90%. Recent papers have focused on the rationalized use of NMBAs, monitoring depth of NMB during anesthesia and the adequate timing and dosing of reversal agents [2], and there are ongoing discussions about the possible benefit of sugammadex over neostigmine, with regards to respiratory outcome [13, 19]. All patients in the shallow/sugammadex group had a return of TOFR to unity (100%), whereas some subjects in the moderate/neostigmine and moderate/sugammadex group had a TOFR in between 90 and 100%. Some of the results may be explained by the combination of repeated doses of neuromuscular blocking agent and a TOFR between 90 and 100%.

Acceleromyography has its imperfections in clinical practice. Even though it is a commonly used method to monitor the depth of neuromuscular blockade, it has its limits in detecting residual neuromuscular blockade in airway muscles [20]. In our study, intercostal muscle activity was reduced at a TOFR of ≥90% but below 100% in patients after moderate neuromuscular blockade and neostigmine administration, compared to the shallow/saline group. These findings further support the return of TOFR to unity after neuromuscular blockade to improve respiratory function if measured with acceleromyography.

Most of the inspiratory work of breathing is performed by the diaphragm, and decreased diaphragm activity is associated with the development of atelectasis [21]. This implies that adequate diaphragm activity is essential in the immediate postoperative period, with its potential benefit being the largest in patients with different coexisting conditions that are associated with diaphragm dysfunction (e.g. underlying heart and lung disease) [22].

Some limitations should be taken into account for this study. Most importantly, we measured EMG activity, which does not necessarily correlate to clinically relevant respiratory outcome values. All patients were successfully extubated and did not have desaturation events in the immediate postoperative period. In a previous study, we found that the healthy male volunteers with lower EMGdi values also had lower PaO_2_ values and tidal volumes [8], but whether the observed decrease in intercostal muscle activity has any clinical relevance remains unclear – i.e. patients among all groups may have sufficient respiratory reserve with the applied NMB strategies. True individual baseline EMG values are lacking in our study, as movement artifacts hindered correct measurements prior to sedation. Secondly, our setup had its technical limitations, as we did not measure tidal volumes or blood gas values, which could have helped in the interpretation of the data. The processing of the EMG data was a very labor-intensive and time-consuming activity, which makes replicating this trial not easy. Furthermore, we had a very large number of excluded patients prior to analysis, which we had to allow if we wanted to get high quality data suitable for analysis. There were multiple reasons for the lack of data quality in these excluded subjects: movement artifacts resulted in high EMG values that could not be filtered from diaphragm activity, removal of surgical drapes dislocated the skin electrodes, and some subjects could be extubated immediately after the return of spontaneous breathing activity, limiting the available amount of data suitable for analysis. Thirdly, as there are different ways of EMG recording and there is a paucity of available recent data in this field of research, we lack baseline values. However, signal intensity can be easily compared in between subjects or groups when using exactly the same standardized method of EMG recording in all subjects, as we did. Furthermore, tests of our setup in healthy volunteers preceding this trial yielded EMG values during resting tidal breathing that were similar to our results (3 to 8 μV). Fourth, we did not include a group that received anesthesia without NMBA administration, which would have served as the ideal control group.

## Conclusions

We compared the effect of different reversal strategies of neuromuscular blockade on EMG parameters when emerging from anesthesia. When compared to a shallow NMB with sugammadex reversal, patients receiving moderate level of NMB had lower diaphragm muscle EMG activity at the moment of liberation from the ventilator. Patients who received a moderate level of NMB and neostigmine had lower intercostal muscle EMG activity compared to those who had a shallow level of NMB and no reversal. These data support the evidence that different levels of NMB and their reversal strategies can have an impact on respiratory outcome after surgery. More evidence on the effects of repeated doses of NMBAs and a full return (to unity) of neuromuscular function on respiratory outcome is needed.

## Supplementary information

**Additional file 1.** Expanded CONSORT flowchart. NMB: neuromuscular block; TOF: train-of-four.

## Data Availability

The datasets used and/or analyzed during the current study are available from the corresponding author on reasonable request.

## Abbreviations

EMG: Electromyographic activity
EMGdi: Electromyographic activity of the diaphragm
IQR: Interquartile range
NMB: Neuromuscular blockade
NMBA: Neuromuscular blocking agent
PTC: Post-tetanic count
TOF: Train-of-four
