# Supplementary figures and images for: Respiratory muscle activity after spontaneous, neostigmine- or sugammadex-enhanced recovery of neuromuscular blockade: a double blind prospective randomized controlled trial
Source: BMC Anesthesiol. 2019 Oct 19;19:187. doi: 10.1186/s12871-019-0863-y (PMC6800991; doi:10.1186/s12871-019-0863-y)

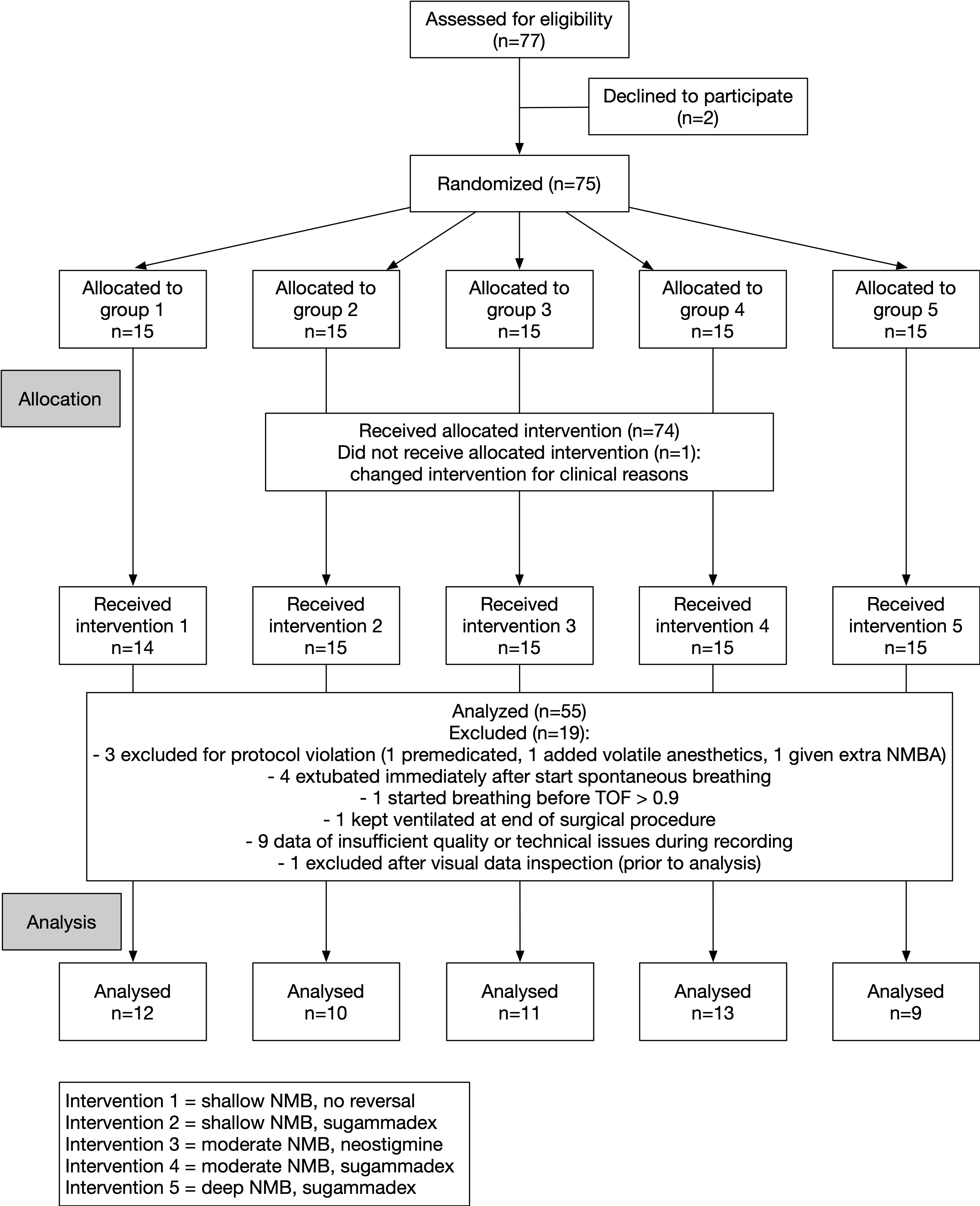

Supplement: Supplementary file 1 — Additional file 1. Expanded CONSORT flowchart. NMB: neuromuscular block; TOF: train-of-four. [file 12871_2019_863_MOESM1_ESM.jpg]
